# The human oncoprotein MDM2 induces replication stress eliciting early intra-S-phase checkpoint response and inhibition of DNA replication origin firing

**DOI:** 10.1093/nar/gkt944

**Published:** 2013-10-24

**Authors:** Rebecca A. Frum, Shilpa Singh, Catherine Vaughan, Nitai D. Mukhopadhyay, Steven R. Grossman, Brad Windle, Sumitra Deb, Swati Palit Deb

**Affiliations:** ^1^Division of Hematology, Oncology and Palliative Care, Department of Medicine, The Massey Cancer Center, Virginia Commonwealth University, Richmond, VA 23298, USA, ^2^Department of Biochemistry and Molecular Biology, The Massey Cancer Center, Virginia Commonwealth University, Richmond, VA 23298, USA, ^3^Department of Biostatistics, The Massey Cancer Center, Virginia Commonwealth University, Richmond, VA 23298, USA and ^4^Department of Medicinal Chemistry, The Massey Cancer Center, Virginia Commonwealth University, Richmond, VA 23298, USA

## Abstract

Conventional paradigm ascribes the cell proliferative function of the human oncoprotein mouse double minute2 (MDM2) primarily to its ability to degrade p53. Here we report that in the absence of p53, MDM2 induces replication stress eliciting an early S-phase checkpoint response to inhibit further firing of DNA replication origins. Partially synchronized lung cells cultured from p53−/−:MDM2 transgenic mice enter S phase and induce S-phase checkpoint response earlier than lung cells from p53−/− mice and inhibit firing of DNA replication origins. MDM2 activates chk1 phosphorylation, elevates mixed lineage lymphoma histone methyl transferase levels and promotes checkpoint-dependent tri-methylation of histone H3 at lysine 4, known to prevent firing of late replication origins at the early S phase. In the absence of p53, a condition that disables inhibition of cyclin A expression by MDM2, MDM2 increases expression of cyclin D2 and A and hastens S-phase entry of cells. Consistently, inhibition of cyclin-dependent kinases, known to activate DNA replication origins during firing, inhibits MDM2-mediated induction of chk1 phosphorylation indicating the requirement of this activity in MDM2-mediated chk1 phosphorylation. Our data reveal a novel pathway, defended by the intra-S-phase checkpoint, by which MDM2 induces unscheduled origin firing and accelerates S-phase entry of cells in the absence of p53.

## INTRODUCTION

Although deregulation of DNA replication is a crucial event in oncogenesis, whether or how oncogenes modulate DNA replication has not been investigated in depth. Several oncogenes that overexpress in cancer cells often induce a growth-suppressive effect in non-transformed cells (1,2), which is considered to be a fail-safe response to suppress uncontrolled cell proliferation. Oncogenic Ras is known to induce a DNA damage repair response (3); Raf-1 induces cell cycle arrest and senescence (4); and MYC is known to trigger DNA damage and checkpoint response (5,6). Furthermore, signs of oncogene-induced senescence are frequently observed in pre-malignant lesions of humans and animal tumors (7). Although the mechanism of Ras-induced DNA hyper-replication leading to senescence has been reported, whether or how other oncogenes influence DNA replication to elicit DNA damage response is largely unknown.

The human homolog of the mouse double minute2 (*mdm2*) gene is known to code for an oncoprotein. Artificial amplification of *mdm2* gene or overexpression of MDM2 in transgenic mice induces tumorigenesis (8,9). The oncoprotein is often overexpressed in human sarcomas and carcinomas in the presence or absence of wild-type (WT) p53 (10,11). MDM2 interacts with the transactivation domain of p53 inactivating its function (12,13). MDM2 is also an E3 ubiquitin ligase, and is known to degrade p53 (14). Although MDM2 interacts physically and functionally to several growth suppressors, such as the retinoblastoma susceptibility gene product (Rb) and p14, its p53-inactivating and degrading function is thought to be the primary cause of oncogenesis (10,13). However, cancer cells with p53 mutation often overexpress MDM2, and the significance of MDM2 amplification or overexpression in human tumors lacking WT p53 is not clear (11,15,16).

Despite its oncogenic function, elevation of MDM2 expression induces G1-arrest in the presence or absence of WT p53 (17–19). Elimination of the growth inhibitory domains of MDM2 rescues its tumorigenic potential (17). Furthermore, in apparently normal cells, such as early passage mouse embryo fibroblasts or limited passage human lung fibroblast (such as WI38), MDM2 inhibits expression of cyclin A (20). Genetic defects, such as absence of WT p53, the cyclin-dependent kinase inhibitor p16 or the transcription factor BRG1, that deregulate the timely expression of cyclin A, also abrogate the ability of MDM2 to inhibit expression of cyclin A expression, but not its ability to induce G1 arrest (17,20), suggesting that MDM2 expression restricts an event downstream of cyclin A expression, and leads us to investigate how MDM2 controls initiation of DNA replication.

Initiation of DNA replication takes place at DNA replication origins, recognized by loading of pre-replication complex during late mitosis and G1 phase, a process known as ‘licensing’. During G1/S transition and at different times during the S phase, replication initiation factors are recruited to only a fraction of licensed origins forming pre-initiation complex, activating the minichromosome maintenance proteins 2–7 (MCM2–7) helicases and assembly of other replication factors and inducing local DNA unwinding. Origin firing is activated by cyclin-dependent kinases complexed with cyclins E and A and by cdc7/DBf4 (21). In mammalian cells, replication origin firing is regulated by checkpoint kinases, ATR and chk1, during normal unperturbed S phase in response to the single-stranded DNA exposed at replication forks (22–24).

In this report, we present evidence to show that elevated expression of MDM2 in cells lacking p53 elevates endogenous levels of cyclins D2 and A and hastens S-phase entry and activation of chk1 phosphorylation. Activation of chk1 phosphorylation is known to stabilize a histone methyl transferase coded by the mixed lineage lymphoma (MLL) gene, which methylates lysine 4 of histone H3 (H3k4) at late replication origins to delay DNA replication (25). Our data show that MDM2 overexpression follows a similar course of events activating chk1 phosphorylation, leading to accumulation of MLL histone methyl transferase and checkpoint-dependent tri-methylation of H3K4 (H3K4Me3) causing inhibition of origin firing. We also show that the MDM2-mediated checkpoint activation is dependent on activation of DNA replication origins by cyclin-dependent kinases. Inhibition of this step using a specific inhibitor of cyclin-dependent kinases abrogates activation of chk1 phosphorylation by MDM2 at the onset of S phase. These data signify that in cells overexpressing MDM2, compromised checkpoint activity would enhance S-phase entry and untimely origin firing, which are known to induce genomic instability (24,26,27).

## MATERIALS AND METHODS

### Plasmids and lentiviral vectors

The ecdysone-inducible plasmid vector system was purchased from Invitrogen, and plasmids expressing MDM2 were constructed by inserting MDM2 cDNA at the multiple cloning site as described earlier (28). Generation of plasmids and lentiviral vectors (pLKO.1) expressing short hairpin (sh) RNA against MDM2 or non-endogenous green fluorescence protein (GFP) from U6 promoter and harboring a puromycin resistance gene has been described earlier (20,29).

### Generation of p53−/−, MDM2 transgenic mice

p53−/−:MDM2 transgenic (p53−/−:MDM2Tr) mice were generated by crossbreeding p53 +/−, MDM2 transgene +/− mice [gift from Stephen Jones, (30) as described earlier (29)].

### Cells, transfections and generation of stable transfectants

H1299 cells were purchased from ATCC and were maintained in media as suggested by the suppliers. H1299 cells expressing MDM2 from ecdysone-inducible promoter were generated following supplier’s (Invitrogen) protocol. The ecdysone-inducible H1299 cells expressed 3–4-fold higher MDM2 transcripts after 24 h of induction with ponasterone. Generation of stable transfectants and cultured lung cells from littermate MDM2 transgenic and non-transgenic mice with p53−/− background has been described earlier. Quantitative polymerase chain reaction (QPCR) analysis of genomic DNA of these cells showed a 10-fold higher copy number of the MDM2 gene than littermate non-transgenic mice (29). To generate stable transfectants expressing shRNA against MDM2 or GFP, appropriate cells were infected with lentiviral vectors expressing respective shRNA. Infected cells were selected with appropriate antibiotics and desired colonies were expanded after analysis.

### Chemicals and drugs

Iododeoxyuridine (IdU), chlorodeoxyuridine (CldU) and caffeine were purchased from (Sigma). Caffeine was used at a concentration of 2 mM for 30 min (31,32), Ponasterone A (Pon; Invitrogen) was used at 1 mM concentration for 24 h as suggested by the supplier and PHA767491 (Tocris Bioscience) was used at a concentration of 10 μM for 60 min (33,34).

### Western blot analysis

Western blot analysis was performed as described previously (35).

### Identification of DNA replication origin firing

**Figure 1.**
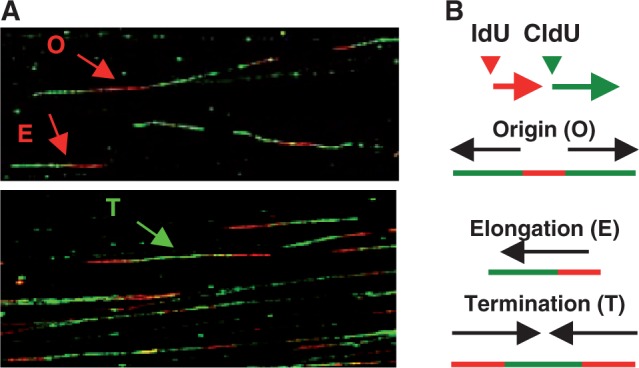
An example of fiber images generated from sequential labeling of replicating DNA by IdU and CldU in H1299 cells and immunostaining with fluorescently labeled antibodies. The figure shows (**A**) classification of the labeled fibers as newly initiated bidirectional origin (O), elongation (E) or termination (T) indicated by arrows along with a cartoon (**B**).

DNA replication origin firing was determined by DNA fiber spreading analysis as described earlier (32). Briefly, cells were pulse-labeled sequentially with the nucleotide analogs IdU (40 μM) and CldU (100 μM) to track the replication pattern and directionality of fork movement. Cells were collected by trypsinization, and genomic DNA of ∼600 cells was aligned on slides by fiber spreading as described earlier. Slides were then air-dried and fixed in 3:1 methanol/acetic acid and dried overnight. After acid treatment (2.5 N HCl, 30 min) and blocking [2% bovine serum albumin (BSA) in phosphate buffered saline (PBS)], DNA fibers on slides were immunostained with primary antibodies against IdU and CldU followed by fluorescently labeled secondary antibodies, and finally fluorescently labeled tertiary antibodies, washed and mounted in antifade. Images were collected by confocal microscopy (Zeiss, LSM700). Newly initiated single origins were detected as red track flanked on both sides by green track as explained in Figure 1A and B. Approximately 200–500 fibers from each sample were scored, and then analyzed using Image J software (NIH).

### Statistical analysis

Distribution of four types of fibers classified as bidirectional origin, origin clusters, elongating forks and terminating forks generated by sequential labeling of replicating DNA over different samples in each experimental set has been tested for independence with Fisher exact test, or by chi-square test in case the computation of Fisher exact test was not feasible due to computer limitations. The null hypothesis of these tests is that the distribution remains independent of the categories, and a significant *P* < 0.05 indicates departure from the hypothesis of independence. All statistical analyses were done using the statistical software R v2.13.0.

### Detection of S-phase nuclei

For detection of S-phase nuclei, density-arrested cells were replated on coverslips and labeled with 40 μM IdU for 20 min at desired time. IdU was washed off using PBS. Cells were fixed with 3% paraformaldehyde solution. For antibody staining, the cells were treated for 5 min with 0.5% triton X-100, followed by 1 h treatment with 2.5 N HCl to denature the DNA. The acid was neutralized with three washes of 0.1 M sodium borate. Cells were then washed with 0.1% Tween in PBS (wash buffer) and blocked in 2% BSA in wash buffer for an hour. Primary antibody against IdU was diluted in 1% BSA in wash buffer, added to coverslips and incubated for an hour. Following three washes with wash buffer, the cells were incubated with Alexafluor 594-conjugated secondary antibody diluted in 1% BSA in wash buffer for an hour. The coverslips were then washed and mounted on slides with Prolong Gold Antifade with DAPI (4′, 6′-diamidino-2-phenylindole hydrochloride, Molecular Probes) and imaged using confocal microscopy (Zeiss LSM700).

### Antibodies

Antibodies against MDM2 were chosen depending on the species. N-20 and SMP14 (Santa Cruz) were used following supplier’s protocol. The 2A10 antibody was a gift from Arnold Levine. Antibodies against Erk2, chk1, cyclin A and cyclin D2 were from Santa Cruz Biotechnology; phospho chk1 (p-chk1) was from Cell Signaling Technology; and MLL^C180^, H3K4Me3 and H3 were from Millipore, and were used following manufacturer’s protocol.

IdU was detected by mouse anti-bromodeoxyuridine (BrdU; Becton Dickinson) primary antibody, and Alexafluor 594-conjugated rabbit anti-mouse (Molecular Probes) and Alexafluor 594-conjugated goat anti-rabbit (Molecular Probe) secondary and tertiary antibodies. CldU was detected by rat anti-BrdU (Accurate) primary antibody, and Alexafluor 488-conjugated chicken anti-rat (Molecular Probe) and Alexafluor 488-conjugated goat anti-chicken (Molecular Probes) secondary and tertiary antibodies as described earlier (32).

### RNA extraction, generation of cDNA and QPCR

Total RNA was isolated from exponentially growing cultured cells using TRIzol reagent (Life Technologies, Invitrogen) following a protocol supplied by the manufacturer. cDNA was synthesized using the Thermoscript reverse transcription-PCR system (Invitrogen). QPCR was performed using a LightCycler system (Roche). Primers were designed using OLIGO 5 software (Molecular Biology Insights) and were synthesized by Integrated DNA Technologies. Reactions were performed in triplicate using SYBR green dye, which exhibits a higher fluorescence upon binding of double-stranded DNA. The methods have been described previously (20). Reactions were performed in triplicate. The QPCR primers used were as follows: (i) MDM2 (human), 5′-TGG CGT GCC AAG CTT CTC TGT- 3′ and 5′-ACC TGA GTC CGA TGA TTC CTG CT- 3′; (ii) GAPDH (human), 5′-GTC AAC GGA TTT GGT CGT ATT-3′ and 5′- GAT CTC GCT CCT GGA AGA TGG-3′, (iii) Cyclin D2 (human) – 5′- TGG GGA AGT TGA AGT GGA AC -3′ and 5′- ATC CAC GTC TGT GTT GGT GA -3′, (iv) MDM2 mouse – 5′ AGG GGA AAG ATA AAG TGG AA -3′ and 5′- ATA AAC AAT GCT GCT GGA AG -3′, and (v) Cyclin D2 (mouse) – 5′- AAA TGG GGG CAG ATG GAG A -3′ and 5′- GGC AAA GGA GGA AGG CGT AT -3′.

## RESULTS

### MDM2 inhibits firing of DNA replication origins

We have previously reported that elevated expression of MDM2 induces G1 arrest in cells irrespective of its p53 status (17,20,36). In apparently normal cells, MDM2 inhibits expression of cyclin A (20). In cells lacking WT p53, MDM2 does not inhibit cyclin A expression, but induces arrest at the G1-S transition (20,36). This observation led us to investigate how MDM2 controls the firing of DNA replication origin, an event downstream of cyclin A expression.

**Figure 2.**
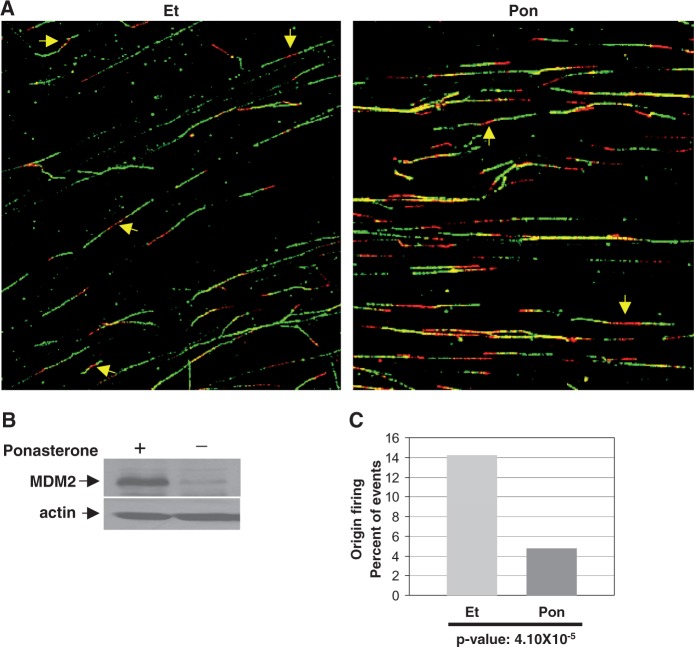
MDM2 inhibits firing of DNA replication origins**.** The figure shows fiber analysis of replicating DNA from H1299 cells that express MDM2 when induced by Pon for 24 h, sequentially pulse labeled for 10 min by IdU and 20 min by CldU and immunostained with fluorescently labeled antibodies. Representative images of labeled DNA fibers from Et- or Pon-treated H1299 cells (**A**) are shown. Arrows show newly fired single origins. Expression of MDM2 in Pon or ethanol-treated cells was determined by western blot analysis (**B**). Actin was used as a loading control. Percentages of bidirectional single origins in Pon or Et-treated cells are shown by bar graphs (**C**). Two hundred to 500 fibers were scored for each sample. *P*-values are indicated at the bottom of the bar graphs.

Because the cyclin A inhibitory function of MDM2 is disabled in H1299 cells (20), we constructed H1299 cells that express MDM2 from an ecdysone-inducible promoter. After treatment with Pon for 24 h, the cells were pulse-labeled sequentially with the nucleotide analogs IdU and CldU to track the replication pattern and directionality of fork movement. Cellular genomic DNA was then aligned on slides by fiber spreading following established techniques (32,37). Incorporated IdU and CldU in the DNA fibers were detected using red or green fluorescent-tagged antibodies, respectively, and visualized by confocal microscopy (Figures 1A and 2A). Newly initiated origins are indicated by a red track flanked on both sides by green, since these patterns were obtained as a consequence of replication origin initiating during the first pulse detected by an antibody tagged with red fluorescence and replication forks moving bidirectionally during the second pulse detected by an antibody tagged with green fluorescence. Similarly, unidirectional red and green tracks indicated elongation, and merging forks were detected by merging green tracks flanked by red tracks as demonstrated in Figure 1. Scoring of 200–400 stained untangled DNA fibers from Pon or ethanol (Et) (vehicle)-treated cells, as shown in the representative images (Figure 2A), revealed that induction of MDM2 expression (Figure 2B) significantly reduced the frequency of origin firing in repeated experiments (Figure 2C, *P*-value = 4.104 × 10^−^^5^). This observation indicates that MDM2 controls the frequency of bi-directional single origin firing. A control H1299 cell line constructed to express the ecdysone inducer and a vector plasmid not harboring MDM2 did not show change in origin firing after treatment with Pon for 24 h (Supplementary Figure S1)

### MDM2-mediated inhibition of origin firing can be rescued by caffeine, an inhibitor of checkpoint kinases

**Figure 3.**
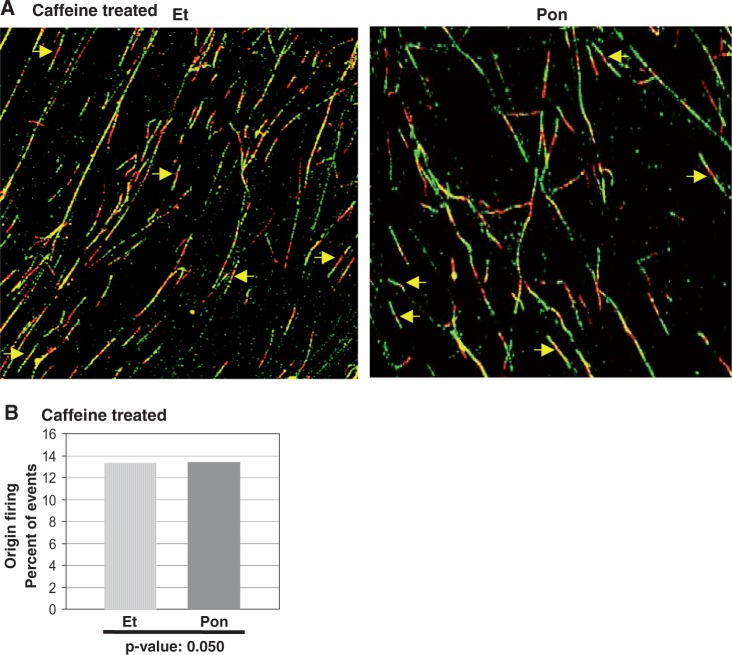
MDM2-mediated inhibition of origin firing can be rescued by caffeine**.** The figure shows fiber analysis of replicating DNA from H1299 cells induced to express MDM2 with Pon for 24 h, followed by treatment with caffeine for 30 min and sequentially pulse-labeled with IdU and CldU as described earlier in the text. Representative images of labeled DNA fibers are shown (**A**). Arrows indicate newly fired single origins. Percentages of bidirectional single origins are shown by bar graphs (**B**). Three hundred to 600 fibers were scored for each sample. *P*-values are indicated at the bottom of the bar graphs.

To test whether inhibition of origin firing by MDM2 is a result of a checkpoint response often elicited by oncogenes (2,5), the ecdysone-inducible H1299 cells were induced to express MDM2 by treatment with Pon for 24 h, followed by a brief (30 min) treatment with caffeine, an inhibitor of ATM or ATR kinases (38,39). Cells were then processed for fiber spreading analysis of replicating DNA as described earlier in the text. Scoring of stained untangled DNA fibers (Figure 3A) showed that Pon and Et-treated cells had a similar frequency of origin firing after caffeine treatment (Figure 3B). The *P-*value (0.050) indicated no significant difference in origin firing between Pon and Et-treated cells. These data show that MDM2-mediated inhibition of origin firing at 24 h was rescued by caffeine treatment, and suggest that inhibition of DNA replication origin firing by MDM2 could be a consequence of a checkpoint response, as they can be rescued by caffeine, an inhibitor of ATM or ATR kinases.

### MDM2 inhibits firing of DNA replication origins at the onset of S phase, and the inhibition can be rescued by caffeine

**Figure 4.**
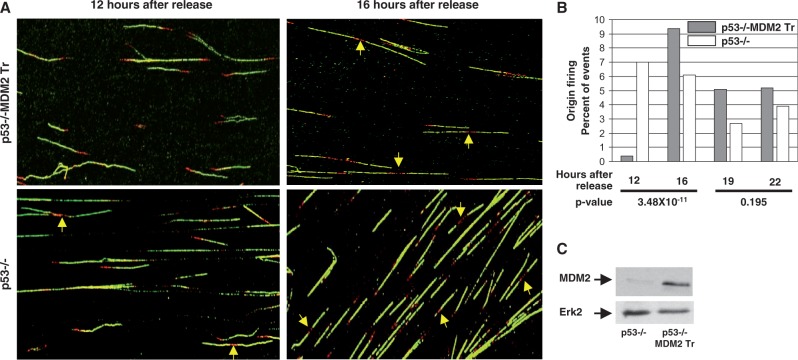
MDM2 inhibits firing of DNA replication origins at the early S phase. The figure shows fiber analysis of replicating DNA from cultured lung cells of p53−/− and p53−/−:MDM2Tr mice sequentially pulse-labeled by IdU for 10 min and CldU for 20 min at different times after replating of density-arrested cells. Representative images of labeled DNA fibers at 12 and 16 h after replating are shown (**A**). Arrows indicate newly fired single origins. The bar graphs show the percentage of bidirectional single origins at different time intervals after replating (**B**). Approximately 150–250 labeled fibers were scored for each sample. *P*-values are indicated at the bottom of the bar graphs. Expression of MDM2 was determined by western blot analysis (**C**). Erk-2 was used as a loading control.

Because MDM2 reduced the frequency of origin firing in ecdysone-inducible H1299 cells (Figure 2), we determined whether MDM2 inhibits origin firing at the early or late S phase. As reasoned above, we determined the cyclin A-independent replication inhibitory function of MDM2 using lung cells isolated from littermate p53−/− and p53−/−:MDM2Tr mice. Cells were partially synchronized by density arrest and replating. The time of onset of the S phase was determined by pulse labeling with BrdU and identifying labeled nuclei at different time intervals. Fiber analysis of replicating DNA from these cells (Figure 4) was performed at different time intervals after release from contact inhibition, as described above. Scoring of untangled labeled fibers (Figure 4A) revealed that, in comparison with p53−/− lung cells, the frequency of origin firing was drastically reduced in p53−/−:MDM2 transgenic lung cells at 12 h after release of contact inhibition. This difference in origin firing not only disappeared but also exceeded the frequency of fired origins in p53−/− cells at 16 h (Figure 4B, *P*-value = 3.479 × 10^−^^11^). Analysis at subsequent time points did not show a significant difference in origin firing between the two cell types (*P*-value = 0.195). Expression of MDM2 was confirmed by western blot analysis (Figure 4C). These data suggest that MDM2 inhibits origin firing at the onset of S phase, and this inhibition disappears at a later time point.

**Figure 5.**
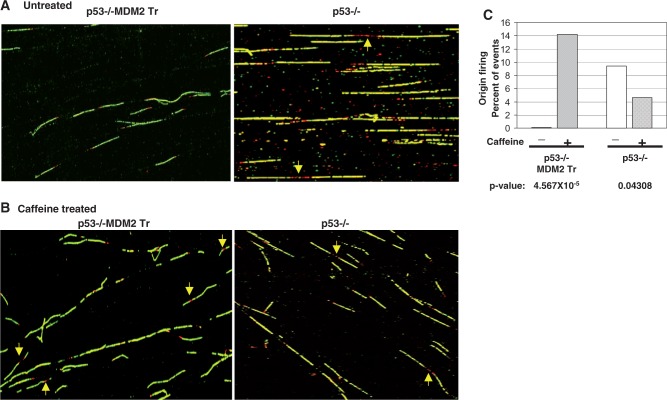
Inhibition of origin firing by MDM2 can be rescued by caffeine. The figure shows fiber analysis of replicating DNA from cultured lung cells of p53−/− and p53−/−:MDM2Tr mice, untreated or treated with caffeine and sequentially pulse-labeled by IdU and CldU at 12 h after replating of density-arrested cells. Representative images of labeled DNA fibers without (**A**) or with (**B**) caffeine treatment at 12 h after replating are shown. Arrows indicate newly fired single origins. The bar graphs show the percentage of bidirectional single origins determined by fiber analysis (**C**). *P*-values are indicated at the bottom of the bar graphs. Approximately 100–200 labeled fibers were scored for each sample.

The transient nature of inhibition of origin firing at the onset of S phase led us to investigate whether MDM2-mediated inhibition of origin firing at the early S phase is a result of an activated checkpoint response. To test this hypothesis, the lung cells from littermate p53−/− and p53−/−:MDM2Tr mice described above were partially synchronized by density arrest and replating. The cells were treated with caffeine, and fiber analysis of replicating DNA was performed at 12 h as described above (Figure 5A and B). Our data showed that at 12 h after replating, inhibition of origin firing was reversed by caffeine treatment in p53−/−:MDM2Tr lung cells, drastically increasing the percentage of fired origins (Figure 5C, *P*-value = 4.567 × 10^−^^5^). Lung cells from littermate p53−/− mice did not show an increase in origin firing at this time point (Figure 5C, *P*-value = 0.04). Thus, elevated levels of MDM2 inhibit origin firing at the onset of S phase, which can be reversed by caffeine, an inhibitor of checkpoint kinases, suggesting an involvement of intra-S-phase checkpoint control in the process.

### MDM2 induces chk1 phosphorylation at the onset of S phase

**Figure 6.**
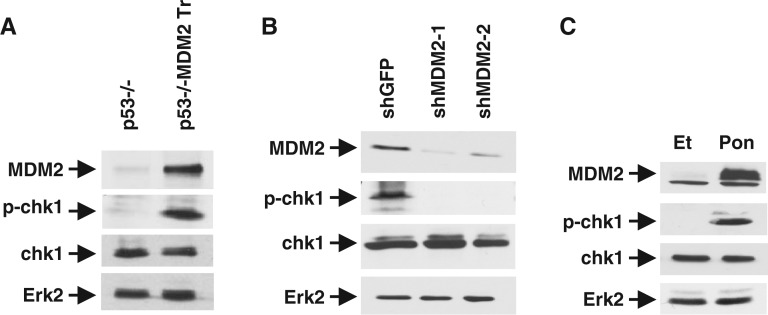
MDM2 induces chk1 phosphorylation at the onset of S phase**.** The figure shows detection of MDM2, p-chk1 and chk1 by western blot analysis of extracts prepared from cells expressing either elevated or knocked-down levels of MDM2. Cultured lung cells of p53−/− and p53−/−:MDM2Tr mice (**A**), and cultured lung cells of p53−/−:MDM2Tr mice stably expressing shRNA against MDM2 (shMDM2-1, shMDM2-2) or GFP (shGFP) (**B**), at 12 h after replating following density arrest were used. Western blot analysis of extracts from H1299 cells expressing MDM2 from an ecdysone-inducible promoter after treatment with Pon or Et for 24 h (**C**) is also shown. Erk-2 was used as a loading control. Migration of MDM2, p-chk1, chk1 and Erk2 is shown by arrows.

As discussed earlier, oncogenes are known to induce a checkpoint response (3–5). As MDM2 is a known oncogene, and it inhibits origin firing at the early S phase, which can be overridden by the checkpoint kinase inhibitor, caffeine (Figures 3 and 5), we investigated whether MDM2 activates the intra-S-phase checkpoint kinase during DNA replication in the absence of WT p53. As described above, matched sets of lung cells isolated from littermate p53−/− and p53−/−:MDM2Tr were partially synchronized by density arrest and replating. Presence of p-chk1 was measured at 12 h after replating by western blot analysis of the cell extracts. Our data indicated that 12 h after replating, p-chk1 levels in lung cells from p53−/−:MDM2Tr mice increased sharply compared with that in the p53−/− mice (Figure 6A).

To confirm that the increased p-chk1 level is due to the presence of elevated levels of MDM2, MDM2 was knocked down in p53−/−:MDM2Tr lung cells. Lung cells from p53−/−:MDM2Tr mice were infected with lentiviral vectors expressing shRNA against MDM2 or GFP control. Cells generated from isolated colonies expressing shRNA against MDM2 or a control shRNA against GFP were partially synchronized by contact inhibition and replating. Twelve hours after replating, p-chk1 levels were determined by western blot analysis. Our data showed that knock down of MDM2 levels drastically diminished p-chk1 levels (Figure 6B).

We also investigated whether elevation of MDM2 expression in H1299 cells leads to activation of chk1 phosphorylation. H1299 cells expressing MDM2 from ecdysone-inducible promoter were induced to express MDM2 with Pon, and p-chk1 levels in the Pon and control (Et-treated) cells were determined by western blot analysis of the cell extracts. Our data indicated that elevation of MDM2 levels by Pon leads to elevation of p-chk1 levels (Figure 6C). Thus, our results indicate that MDM2 can activate phosphorylation of the intra-S-phase checkpoint kinase chk1.

### MDM2 hastens the intra-S-phase checkpoint response

**Figure 7.**
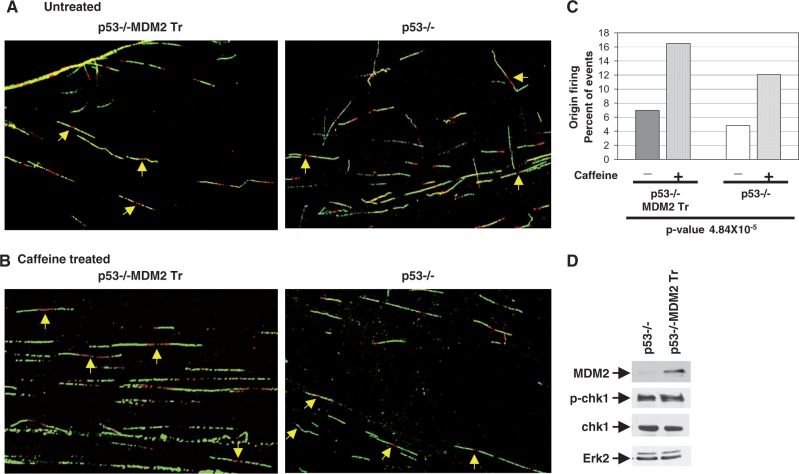
MDM2 hastens intra-S-phase checkpoint response. The figure shows that firing of DNA replication origins is increased by caffeine treatment at 16 h after replating of density-arrested cultured lung cells of both p53−/− and p53−/−:MDM2Tr mice. Representative fiber images of replicating DNA from cultured lung cells of p53−/− and p53−/−:MDM2Tr mice untreated (**A**) or treated with (**B**) caffeine and pulse-labeled at 16 h after replating are shown. Arrows indicate newly fired single origins. The bar graphs show the percentage of bidirectional single origins determined by fiber analysis (**C**). *P*-values are indicated at the bottom of the bar graphs. Approximately 230–270 labeled DNA fibers were scored for each sample. Expression of MDM2 and chk1 phosphorylation at 16 h after replating of the density-arrested p53−/− and p53−/−:MDM2Tr lung cells is shown by western blot analysis of the cell extracts in (**D**, right). Erk-2 was used as loading control. Migration of MDM2, p-chk1, chk1 and Erk2 is shown by arrows.

Checkpoint kinases are known to limit origin firing during normal S-phase progress (40,41). As p53−/−:MDM2Tr lung cells showed a drastic increase in chk1 phosphorylation and inhibition of origin firing compared with p53−/− lung cells 12 h after contact inhibition and replating, we determined whether p53−/− cells activate a checkpoint response at a later time point. For this purpose, p53−/− and p53−/−:MDM2Tr lung cells were contact-inhibited and replated. DNA replication origin firing and chk1 phosphorylation were determined at 16 h after replating of density-arrested cells, as described above. Scoring of labeled DNA fibers (Figure 7A) revealed that, consistent with the experiment shown in Figure 4, a similar percentage of origins was fired in both the cell types (Figure 7A and C). Inhibition of checkpoint-dependent origin firing was evidenced by release of this inhibition by treatment with caffeine to a similar extent (∼2.4-fold) in the two cell types (Figure 7B and C, *P-*value = 4.849 × 10^−^^5^). Consistent with these observations, both the cell types showed chk1 phosphorylation to a similar extent at 16 h after replating (Figure 7D). These data indicate that the p53−/−:MDM2Tr cells induce S-phase checkpoint response earlier than p53−/− cells.

### MDM2 elevates MLL histone methyl transferase levels

Next we wished to determine how early induction of chk1 phosphorylation by MDM2 would lead to inhibition of origin firing. Although the phosphatase CDC25A that controls activation of cyclin-dependent kinases has been implicated in inhibition of origin firing by chk1 phosphorylation (24,40), MDM2 did not alter CDC25A levels in our experiments (data not shown). Alternatively, stabilization of MLL histone methyl transferase has been reported to inhibit origin firing (25). Translocation of the MLL gene has been related to childhood and adult leukemia (42). The gene codes for a precursor protein, which is processed to generate a heterodimer, MLL^N320/C180^. MLL deficiency causes S-phase checkpoint dysfunction (43). MLL contributes to crucial cellular functions by methylating H3K4 by its C-terminal SET domain (44). Intra-S-phase ATR signaling phosphorylates MLL histone methyl transferase (MLL^C180^) preventing its degradation (25). Accumulated MLL^C180^ then methylates H3K4, diminishing CDC45 loading to delay DNA replication (25).

**Figure 8.**
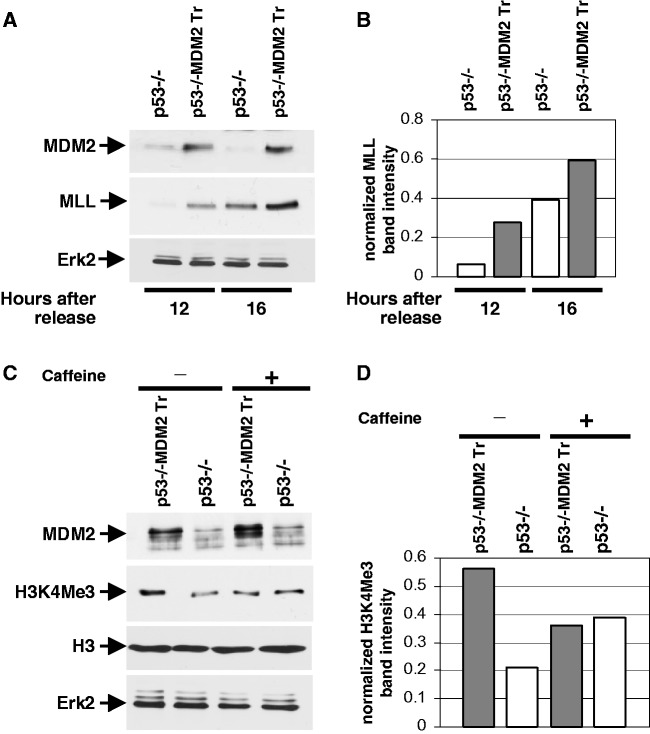
MDM2 elevates MLL histone methyl transferase levels (**A**, **B**) and H3K4 tri-methylation in a checkpoint-dependent manner (**C**, **D**). The figure shows western blot analysis of extracts prepared from cultured lung cells of p53−/− and p53−/−:MDM2Tr mice at 12 and 16 h after replating of density-arrested cells to determine accumulation of MLL histone methyl transferase levels using a specific antibody (**A**), and acid extracts prepared from cultured lung cells of p53−/− and p53−/−:MDM2Tr mice at 12 h after replating of density-arrested cells with or without caffeine treatment to determine H3K4Me3 levels using a specific antibody (C). Erk-2 was used as a loading control in both the experiments. Migration of MDM2, MLL histone methyl transferase (MLL), H3K4Me3, H3 and Erk2 is shown by arrows. Differences in the MLL histone methyl transferase (B) and H3K4Me3 (D) levels were determined by densitometry and are shown by bar graphs at the right of each blot.

Because elevation of MDM2 levels caused chk1 phosphorylation and inhibition of origin firing, we investigated whether MDM2 elevates MLL^C180^ levels at the onset of S phase. We determined MLL^C^ ^80^ levels in extracts prepared from p53−/− and p53−/−:MDM2Tr lung cells at 12 and 16 h after density arrest and replating by western blot analysis as described above. Our data (Figure 8A) showed that compared with p53−/− lung cells, MLL^C180^ levels are remarkably elevated in p53−/−:MDM2Tr lung cells at 12 h. Densitometric analysis (Figure 8B) revealed a 4.5-fold increase in MLL^C180^ levels p53−/−:MDM2Tr lung cells. However, at 16 h, the MLL^C180^ levels also increased in p53−/− lung cells, and the difference in the MLL^C180^ levels in the two cell types was reduced (1.5-fold). This result indicates that presence of MDM2 leads to early accumulation of MLL^C180^.

### MDM2 upregulates H3K4 tri-methylation in a checkpoint-dependent manner

Early accumulation of MLL^C180^ in p53−/−:MDM2Tr lung cells led us to investigate whether these cells show enhanced tri-methylation of H3K4 (H3K4Me3), and whether increase in H3K4Me3 levels is checkpoint-dependent. Acid extraction of the genomic DNA of p53−/− and p53−/−:MDM2Tr lung cells harvested 12 h after density arrest and replating followed by western blot analysis revealed higher H3K4Me3 levels in p53−/−:MDM2Tr than p53−/− lung cells (Figure 8C). Densitometric analysis (Figure 8D) revealed a >2.5-fold increase in the H3K4Me3 levels in p53−/−:MDM2Tr compared with p53−/− lung cells. To determine whether the increase in H3K4Me3 levels is due to checkpoint activation by MDM2, p53−/− and p53−/−:MDM2Tr lung cells were treated with caffeine before harvesting at 12 h. Cells were then harvested and their genomic DNA was subjected to acid extraction. Western blot analysis of the extracts revealed that caffeine treatment abrogates the increase in H3K4Me3 levels in p53−/−:MDM2Tr lung cells (Figure 8C and D). These data indicate that inhibition of checkpoint activation in cells with elevated levels of MDM2 abrogates the increase in tri-methylation of H3K4.

### MDM2 upregulates cyclin D2 and cyclin A expression in the absence of p53

**Figure 9.**
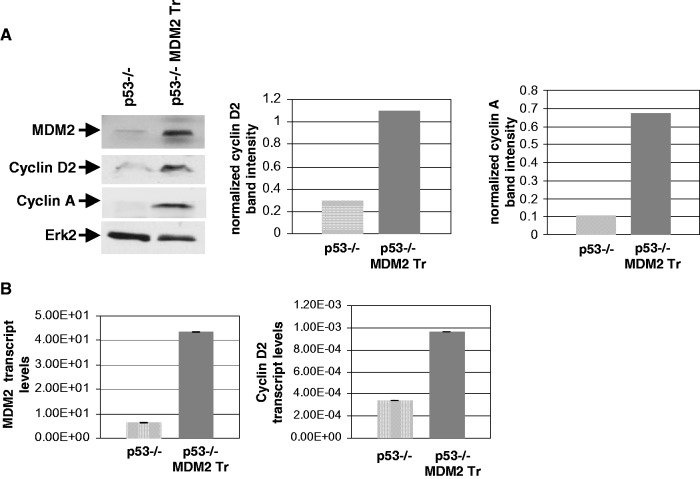
MDM2 increases expression of cyclin D2 and cyclin A in cultured lung cells from p53−/− and p53−/−:MDM2Tr mice. The figure shows MDM2 and endogenous cyclin D2 and cyclin A protein levels (**A**) and MDM2 and cyclin D2 transcripts (**B**) in lung cells of p53−/− and p53−/−:MDM2Tr mice determined by western blot analysis and QPCR, respectively. Erk2 was used as a loading control in western blot analysis. Arrows indicate migration of MDM2, cyclin D2, cyclin A and Erk2. The densitometric analysis of cyclin D2 and cyclin A expression is shown by bar graphs on the right (A), and the transcript levels were normalized by GAPDH expression, and are shown by bar graphs (B).

**Figure 10.**
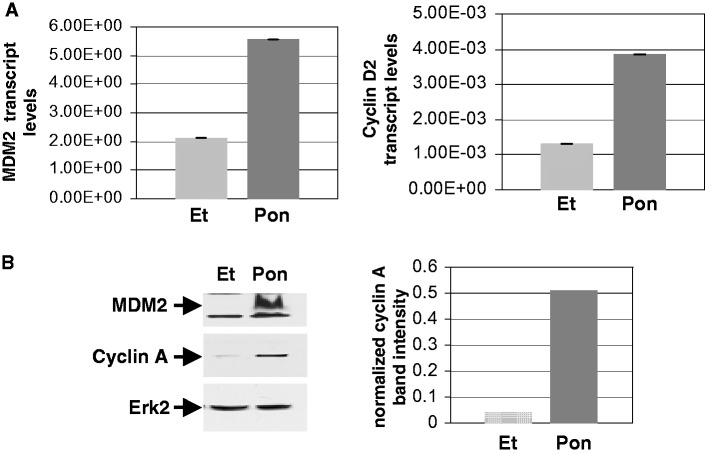
MDM2 increases the expression of cyclin D2 and cyclin A in H1299 lung cancer cells. The figure shows MDM2 and endogenous cyclin D2 transcript (**A**) and cyclin A protein levels (**B**) in H1299 cells expressing MDM2 from an ecdysone-inducible promoter determined by QPCR and western blot analysis, respectively, after treatment with Pon or Et for 24 h. The transcript levels were normalized by GAPDH expression, and are shown by bar graphs (A), and the densitometric analysis of cyclin A expression is shown by bar graphs on the right (B). Erk2 was used as a loading control. Arrows indicate migration of MDM2, cyclin A and Erk2.

We next investigated how MDM2 may activate the intra-S-phase checkpoint in the absence of p53. Although MDM2 inhibits cyclin A expression in normal cells with WT p53 (20), our unpublished gene expression analysis in p53−/− lung cells suggested that MDM2 upregulates expression of cyclin D2 in the absence of WT p53. Overexpression of cyclins is known to induce excessive origin firing causing replication stress (33) and slowing of DNA replication. This information led us to investigate whether MDM2 upregulates the expression of cyclin D2. Therefore, we determined the expression of cyclin D2 in p53−/− and p53−/−:MDM2Tr lung cells both at the level of protein by western blot analysis and RNA by QPCR as described earlier (35). Our data showed elevated levels of cyclin D2 protein (Figure 9A) and RNA (Figure 9B) in p53−/−:MDM2Tr lung cells compared with p53−/− lung cells. Densitometric analysis revealed a > 3-fold increase in the cyclin D2 protein levels, and QPCR data showed ∼3-fold increase in the RNA levels (middle) suggesting that MDM2 upregulates cyclin D2 expression. Induction of MDM2 expression from an ecdysone-inducible promoter using Pon in H1299 cells showed similar data, confirming that MDM2 upregulates cyclin D2 (Figure 10).

Because elevated expression of cyclin D2 should allow crossing of the restriction point of cell cycle and thus should lead to an increase in cyclin A expression, we determined cyclin A protein levels in p53−/− and p53−/−:MDM2Tr lung cells by western blot analysis. Compared with p53−/− lung cells, p53−/−:MDM2Tr lung cells showed a sharp elevation of cyclin A levels (Figure 9A, top right) suggesting that elevation of cyclin D2 levels boosted cell cycle progress. H1299 cells expressing MDM2 from an ecdysone-inducible promoter when induced to express MDM2 with Pon also showed elevated levels of cyclin A (Figure 10B), implicating MDM2 in the accelerated progress toward initiation of DNA replication.

Overexpression of MDM2 in p53-null human osteosarcoma Saos-2 cells, which harbors nonfunctional RB (45,46), increased cyclin D2 expression. However, increase in cyclin A expression and checkpoint phosphorylation by MDM2 were less significant compared with that of Pon-induced H1299 or p53−/− MDM2Tr cells (Figures 6 and 9), suggesting that defects in cyclin D2 downstream pathway desensitizes intra-S-phase checkpoint response (Supplementary Figure S2).

To confirm that increased levels of cyclin D2 expression in p53−/−:MDM2Tr lung cells are due to elevated levels of MDM2, we determined whether knock down of MDM2 in these cells would diminish cyclin D2 expression. Our data showed that knock down of MDM2 decreased cyclin D2 expression both at the levels of RNA and protein (Supplementary Figure S3)

Cyclin D2 is expressed in response to mitogenic signals (47), and PI3-kinase pathway regulates cyclin D2 expression (48). Because MDM2 upregulates PI3-kinase activity(29), we determined whether MDM2-mediated upregulation of cyclin D2 expression is susceptible to a PI3-kinase inhibitor, Wortmannin (49). Our data show that Wortmannin inhibits increase in cyclin D2 expression by MDM2 (supplementary Figure S4), implicating MDM2-mediated activation of PI3 kinase activity in cyclin D2 upregulation by MDM2

### MDM2 hastens S-phase entry of cells

**Figure 11.**
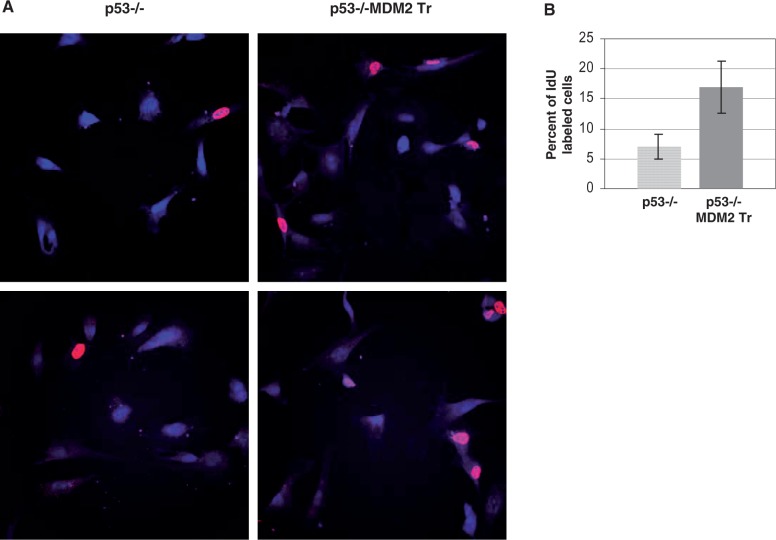
MDM2 hastens S-phase entry of cells. Cultured lung cells of p53−/− and p53−/−:MDM2Tr mice were pulse-labeled by IdU at 12 h after replating of density-arrested cells and immunostained with fluorescently labeled antibodies. Cells were stained with DAPI for detection of nuclei. One hundred fifty to 200 nuclei were scored in each of triplicate sets of experiments. Two representative images of each of labeled p53−/− or p53−/−:MDM2Tr lung cells are shown (**A**). The bar graph (**B**) shows the percentage of IdU-labeled p53−/− or p53−/−:MDM2Tr lung cells at 12 h after replating of density-arrested cells.

Because elevated levels of MDM2 increased cyclin D2 expression, we determined whether the presence of MDM2 accelerated S-phase entry of cells, thus activating the intra-S-phase checkpoint response sooner in p53−/−:MDM2Tr lung cells compared with p53−/− lung cells to prevent further origin firing. We therefore investigated the number of p53−/− and p53−/−:MDM2Tr lung cells entering S phase at 12 h after replating of density-arrested cells. Cells were pulse-labeled with IdU at different time intervals, fixed, acid-treated, stained with a specific antibody and mounted in antifade with DAPI as described in Materials and Methods. Images (Figure 11A) of 150–200 nuclei were collected by confocal microscopy from each sample, and percentage of IdU-labeled nuclei was determined. Our data showed lack of IdU incorporation until 11 h after replating (data not shown). At 12 h, the cells showed IdU incorporation (Figure 11A), and an average of 7% p53−/− lung cells incorporated IdU as opposed to 17% of p53−/−:MDM2Tr lung cells (Figure 11B). These data suggest accelerated S-phase entry of p53−/−:MDM2Tr lung cells compared with p53−/− lung cells at early S phase.

### Inhibition of cyclin-dependent kinases that activate replication origins abrogates MDM2-mediated chk1 phosphorylation

In metazoan cells, origins are activated by cyclin-dependent kinases and cdc7 kinases, which phosphorylate the MCM2–7 activating the MCM helicases and inducing melting of double-stranded DNA at the origin of replication (23,50). Because MDM2 upregulates cyclin D2 and cyclin A expression in cells lacking WT p53, we considered whether an increase in expression of cyclins led to enhanced activation of cyclin-dependent kinases, and thus activation of origins, generating replication stress and intra-S-phase checkpoint signaling. A cdc7 kinase inhibitor PHA767491 has been shown to specifically prevent activation of origins by inhibiting DNA helicases, but not to impede fork progression (33,34).

**Figure 12.**
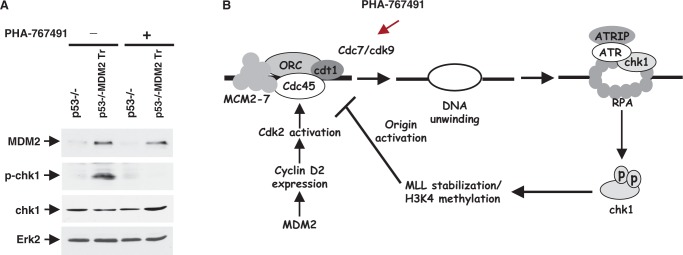
A specific inhibitor, PHA 767491, of cyclin-dependent kinases that activate replication origins abrogates MDM2-mediated chk1 phosphorylation (**A**). Induction of chk1 phosphorylation was determined by western blot analysis of extracts prepared from cultured lung cells of p53−/− and p53−/−:MDM2Tr mice at 12 h after replating of density-arrested cells with or without PHA 767491 (10 μM) treatment for 1 h. Erk-2 was used as a loading control. Migration of MDM2, p-chk1, chk1 and Erk2 is shown by arrows. The proposed mechanism of MDM2-mediated checkpoint response is shown in (**B**, right).

To test whether origin activation by MDM2 induces chk1 phosphorylation, p53−/− and p53−/−:MDM2Tr lung cells were partially synchronized by density arrest and replating; cells were then treated with PHA767491 to prevent origin activation before harvesting at 12 h, and chk1 phosphorylation in the cell extracts was determined by western blot analysis. Our data (Figure 12A) showed that treatment with the cdc7 inhibitor abrogated MDM2-mediated chk1 phosphorylation. These data indicate that an increase in cyclin D2 and consequently cyclin A expression by MDM2 hastens untimely origin activation promoting chk1 phosphorylation at the onset of S phase. Consistently, overexpression of MDM2 in human cancer cell Saos-2 with deleted p53 and inactive RB although upregulates cyclin D2 does not increase cyclin A expression and chk1 phosphorylation significantly (supplementary Figure S2).

## DISCUSSION

Overexpression of oncogenes is a hallmark of cancer, and is widely known to confer growth advantage in cells. On the contrary, several oncogenes that are frequently overexpressed in cancer cells and are used as predictive markers restrain cell proliferation (1–5). According to the established model, the primary cause of oncogenesis mediated by the human oncoprotein MDM2 has been ascribed to its ability to interact with and degrade WT p53. Although this model is well evidenced, it does not explain amplification or overexpression of MDM2 in cancer cells that lack WT p53. In this communication, we report a novel pathway by which MDM2 overexpression leads to unscheduled activation of DNA replication origins in the absence of p53, and a mechanism by which non-transformed cells may combat this function. Loss of this defense mechanism would lead to unscheduled initiation of DNA replication, which has been related to genome instability (24,26,27).

We have reported earlier that MDM2 overexpression induces G1-arrest in the presence or absence of p53 (17,20,36). In apparently normal non-transformed cells, MDM2 inhibits expression of cyclin A. However, it requires p53 for this function (20). Although MDM2 cannot inhibit cyclin A expression in the absence of p53, it induces G1-arrest (17,36). In this report, we show evidence that in the absence of p53, elevated levels of MDM2 induce a checkpoint response and thus inhibit firing of DNA replication origins at the early S phase (Figures 2, 4 and 6) reducing progression of cells from early- to mid-S phase. In flow cytometric analysis, cells in early S phase tend to merge with the G1-peak generating an appearance of inhibition of G1 to S-phase transition. As expected, p53−/− cells, which express low levels of MDM2, exhibit intra-S-phase checkpoint arrest and inhibition of origin firing, but at a later time point than p53−/−:MDM2Tr cells (Figure 7). We have also presented data (Supplementary Figure S5) that MDM2-mediated inhibition of origin firing is not a result of upregulation of cyclin-dependent kinase inhibitor p21 due to stabilization of p73 by MDM2.

We show that an increase in MDM2 expression in the absence of p53 elevates cyclin D2, and cyclin A levels the accelerating S-phase-entry (Figures 9 and 10). D-type cyclins have redundant functions, and are expressed at early G1 (51). Cyclin D2 is activated by mitogenic signals, and is believed to convey extracellular signals that lead to DNA replication and cell proliferation (47). PTEN/GSK3 β pathway regulates cyclin D2 expression (48). Recently, we have shown that MDM2 upregulates PI-3 kinase and GSK3 β phosphorylation. In this communication, we have provided evidence to implicate MDM2-mediated upregulation of PI3-kinase activity in cyclin D2 upregulation by MDM2 (Supplementary Figure S4). The exact mechanism of this transcriptional activation by MDM2 is under investigation at present. Also, several transcription factors including Shh (sonic hedgehog) and Gli are known to participate in cyclin D2 expression (52). It remains to be determined how MDM2 may integrate in these pathways.

Presence of a higher percentage of IdU-labeled p53−/−:MDM2Tr cells compared with p53−/− cells at 12 h after contact inhibition and replating (Figure 11) indicates that cells expressing MDM2 initiate DNA replication earlier than p53−/− cells, and is consistent with elevation of cyclin D2 and cyclin A expression by MDM2. Elevation of cyclin D2 expression by MDM2 is expected to enable the cells to cross the restriction point of the cell cycle, leading to activation of origins. However, acceleration of origin activation (DNA melting) by MDM2 during S-phase entry at 12 h activates a checkpoint response (Figure 6), which allows activated origins to progress as evidenced by pulse-labeled nuclei (Figure 11), but inhibits further origin firing in the cells (Figures 2 and 4). The S-phase checkpoint response restricts origin firing at 16 h in both p53−/− and p53−/−:MDM2Tr cells as evidenced by chk1 phosphorylation and increase in bi-directional single origin firing by caffeine (Figure 7). Consistently, a specific block in origin activation abrogates the MDM2-mediated checkpoint response (Figure 12A). Our proposed model (Figure 12B) depicts this pathway.

Accumulation of MLL histone methyl transferase and consistent increase in H3K4 methylation by MDM2 suggest a mechanism by which the MDM2-mediated checkpoint response would lead to inhibition of origin firing. We also show that MDM2-mediated H3K4 methylation is checkpoint-dependent, as it can be inhibited by caffeine (Figure 8C).

Our data signify that a compromised checkpoint response would allow an increase in untimely origin firing by MDM2. ATR and chk1 kinases are required for cell survival (53), and mutation or inactivation of ATR or chk1 kinases is not a frequent event observed during oncogenesis. However, mutation in the downstream participants, such as translocation of N-terminal 1400 amino acids of MLL, has been observed frequently in leukemia and other cancers (25,42). MLL fusion proteins have been demonstrated to function as dominant negative mutants that abrogate ATR-mediated stabilization of MLL and compromise the S-phase checkpoint (25). Therefore, overexpression or amplification of MDM2, which has also been reported in leukemia and lung cancers (11,54), in cells with compromised MLL histone methyl transferase function could accelerate untimely DNA replication, which is known to induce gene abnormality (24,26,27).

## SUPPLEMENTARY DATA

Supplementary Data are available at NAR Online, including [55].

## FUNDING

Funding for open access charge: This project was funded by a pilot project from VCU Massey Cancer Center (MCC) (to S.P.D.), and NCI [CA 121144 to S.D.]; Supported by pilot project from MCC (to S.P.D. and R.A.F.), and NCI [CA107532 to S.R.G.]. Funding for open access charge will be paid by corresponding author personally.

*Conflict of interest statement*. None declared.

## Supplementary Material

Supplementary Data
