# Supplementary material for: The human oncoprotein MDM2 induces replication stress eliciting early intra-S-phase checkpoint response and inhibition of DNA replication origin firing
Source: Nucleic Acids Res. 2013 Oct 24;42(2):926–40. doi: 10.1093/nar/gkt944 (PMC3902934; doi:10.1093/nar/gkt944)
Supplement: Supplementary Data [file supp_42_2_926__index.html]

The human oncoprotein MDM2 induces replication stress eliciting early intra-S-phase checkpoint response and inhibition of DNA replication origin firing — Supplementary Data 

# The human oncoprotein MDM2 induces replication stress eliciting early intra-S-phase checkpoint response and inhibition of DNA replication origin firing

## Supplementary Data

files

**Files in this Data Supplement:**

- Supplementary Data - pdf file
