# Supplementary material for: The human oncoprotein MDM2 induces replication stress eliciting early intra-S-phase checkpoint response and inhibition of DNA replication origin firing
Source: Nucleic Acids Res. 2013 Oct 24;42(2):926–40. doi: 10.1093/nar/gkt944 (PMC3902934; doi:10.1093/nar/gkt944)
Supplement: Supplementary Data [file supp_gkt944_NAR_supplementary_datav3.pdf]

Supplementary data

Rebecca A Frum, Shilpa Singh, Catherine Vaughan, Nitai D Mukhopadhyay, Steven R Grossman, Brad Windle, Sumitra Deb and Swati Palit Deb. **The human oncoprotein MDM2 induces replication stress eliciting early intra-S phase checkpoint response and inhibition of DNA replication origin firing**

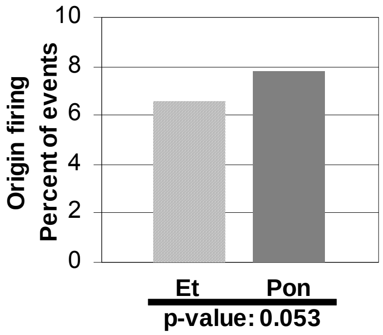

Figure S1: Ponasterone A does not alter origin firing.

To determine the effect of Ponasterone A on firing of DNA replication origin, we constructed a control H1299 cell line harboring a vector plasmid without MDM2 and a plasmid (pvgRxR, Invitrogen) expressing the ecdysone inducer. The control cell line was treated with ethanol (Et) or Ponasterone A (Pon) for 24 hours, and processed for fiber analysis of replicating DNA after sequentially pulse labeling by IdU for 10 minutes and CldU for 20 minutes and immunostaining with fluorescently labeled antibodies. Scoring of approximately 230 labeled fibers from each sample revealed no alteration in the frequency of origin firing after treatment with Ponasterone A for 24 hours (p-value 0.053).

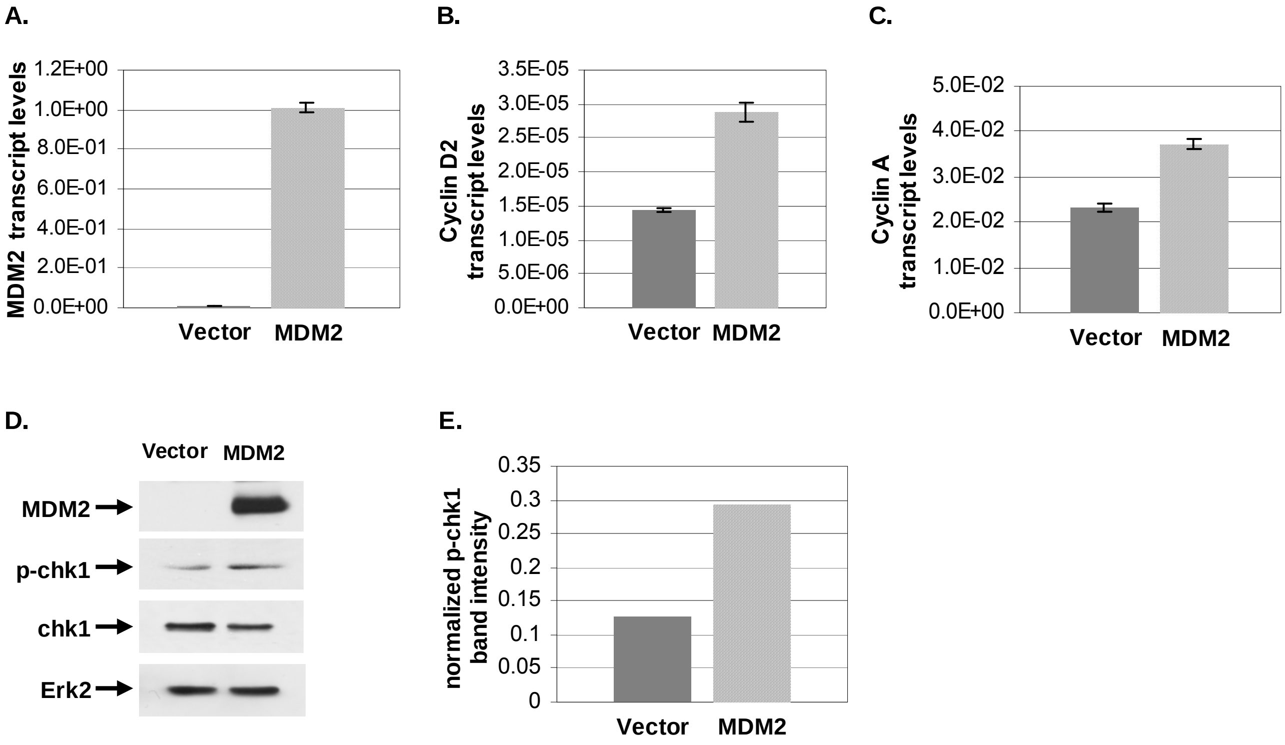

Figure S2: MDM2 overexpression in Saos-2 cells elevates cyclin D2 expression, but not cyclin A or chk1 phosphorylation significantly.

MDM2 expression plasmid or an empty vector (5 μg) was introduced in p53-null human osteosarcoma Saos-2 cells ($3X10^6$), which harbor nonfunctional RB, by nucleofection. Cells were harvested after 12 hours and either processed for RNA extraction and cDNA preparation or for Western blot analysis. MDM2 (A), cyclin D2 (B) and cyclin A (C) transcript levels normalized by GAPDH expression are shown by bar graphs. MDM2, p-chk1, chk1 and Erk2 levels were determined by Western blot analysis (D). Arrows indicate migration of MDM2, p-chk1, chk1 and Erk2. Erk2 was used as a loading control in Western blot analysis. The densitometric analysis of p-chk1 levels is shown by bar graphs on the right (E). The data show that MDM2 increased cyclin D2 expression (B). However, increase in cyclin A expression (C), and checkpoint phosphorylation (D, E) by MDM2 was not significant suggesting that defects in cyclin D2 downstream pathway desensitizes intra-S phase checkpoint response.

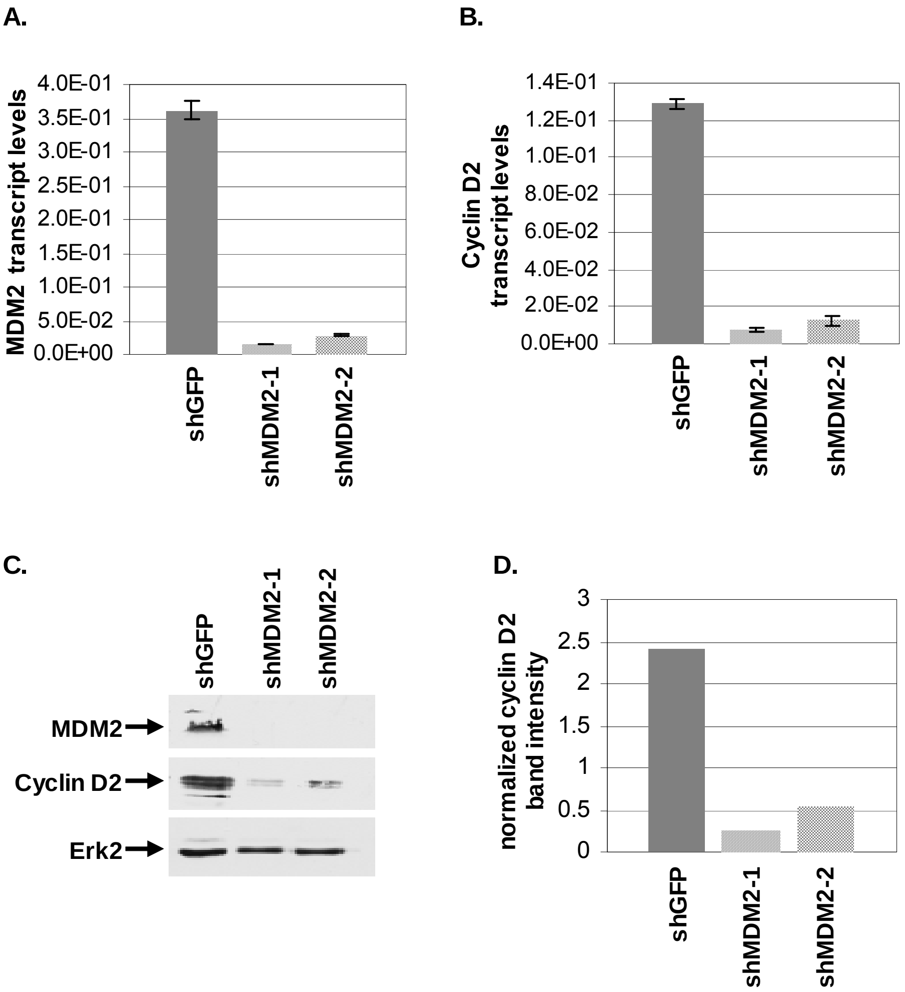

Figure S3: Knockdown of MDM2 decreases cyclin D2 expression drastically.

To confirm that increased levels of cyclin D2 expression in lung cells of p53-/-:MDM2Tr mouse is due to elevated levels of MDM2, we determined if knock down of MDM2 in these cells would diminish cyclin D2 expression. Expression of MDM2 (A) and cyclin D2 (B) transcripts in p53-/-:MDM2Tr lung cells expressing shRNA against MDM2 (shMDM2-1, shMDM2-2) or GFP (shGFP) are shown by bar graphs. Transcript levels were normalized by GAPDH expression. MDM2, cyclin D2 and Erk2 protein expression was determined by Western blot analysis (C). Arrows indicate migration of MDM2, cyclin D2 and Erk2. Erk2 was used as a loading control in Western blot analysis. The densitometric analysis of cyclin D2 protein levels normalized by Erk2 levels is shown by bar graphs on the right (D). Our data showed that knockdown of MDM2 decreased cyclin D2 expression.

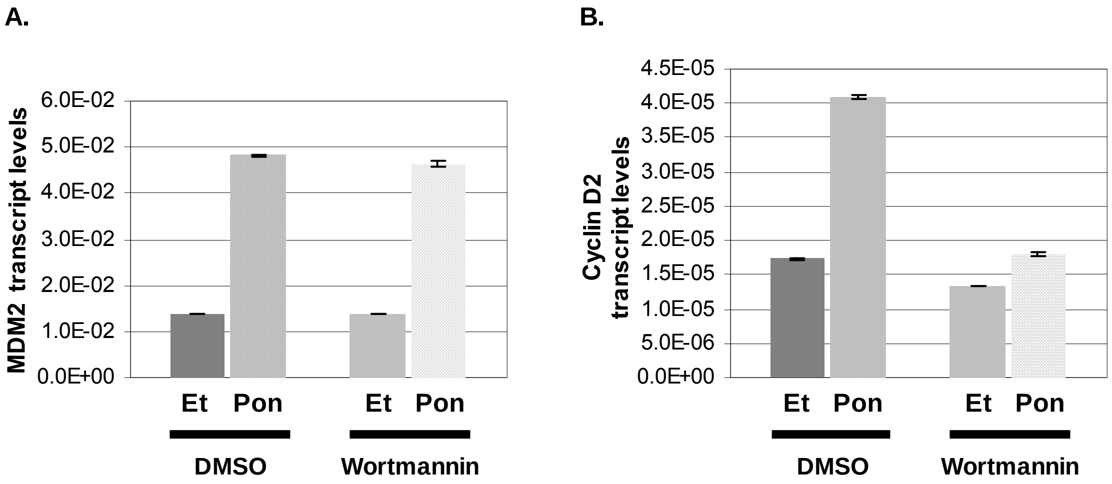

Figure S4: The PI3-kinase inhibitor Wortmannin inhibits MDM2-mediated up-regulation of cyclin D2 expression.

Cyclin D2 is expressed in response to mitogenic signals (1), and PI3-kinase pathway has been shown to regulate cyclin D2 expression (2). Since MDM2 up-regulates PI3-kinase activity (3), we determined if MDM2-mediated upregulation of cyclin D2 expression is susceptible to a PI3-kinase inhibitor, Wortmannin (4). To determine whether wortmannin inhibits an increase in cyclin D2 expression by MDM2, H1299 cells expressing MDM2 from an ecdysone inducible promoter were either induced to express MDM2 by Ponasterone A (Pon) treatment or left uninduced with ethanol (Et) treatment for 24 hours in the presence of Wortmannin (50nM) or DMSO for the last 4 hours. Expression of MDM2 (A) and cyclin D2 (B) transcript levels normalized by GAPDH expression is shown by bar graphs. Our data show that Wortmannin inhibits increase in cyclin D2 expression by MDM2.

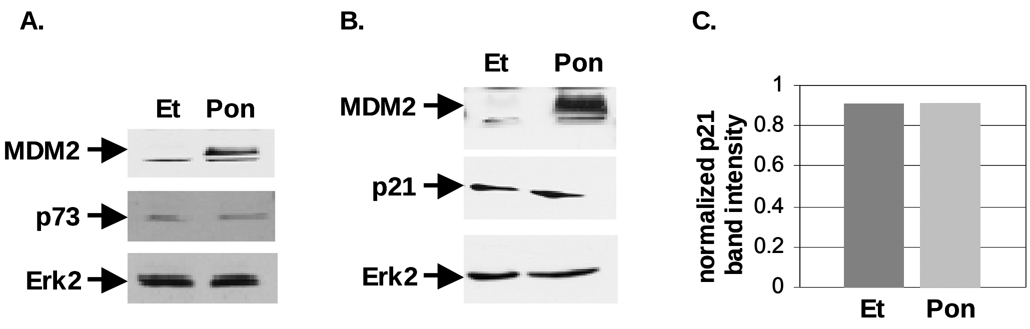

Figure S5: MDM2 does not up-regulate cyclin kinase inhibitor p21 in p53-null H1299 cells:

To exclude the possibility that MDM2-mediated inhibition of origin firing is a result of increase in cyclin dependent kinase inhibitor p21 expression through stabilization of p73, we determined p73 and p21 expression by Western blot analysis in extracts prepared from H1299 cells expressing MDM2 from ecdysone inducible promoter by Ponasterone A (Pon) treatment or left uninduced with ethanol (Et) treatment for 24 hours. Expression of p73 (A), p21 (B), MDM2 and Erk2 protein expression was determined by Western blot analysis. Arrows indicate migration of MDM2, p73, p21 and Erk2. Erk2 was used as a loading control. Densitometric analysis of p21 protein levels normalized by Erk2 levels is shown by the bar graphs on the right (C). Our data showed that MDM2 does not alter p73 or p21 expression levels.

Methods and Materials:

Cells and transfection: H1299 cells expressing MDM2 from an ecdysone inducible promoter and control cells were constructed using ecdysone inducible plasmid vector system from Invitrogen. Saos-2 cells were from ATCC, and were grown following supplier's protocol. Construction of MDM2 expression plasmids and p53-/- MDM2 transgenic murine lung cells expressing shRNA against MDM2 or GFP has been described earlier (3,5). Nucleofection was performed using a nucleofector and nucleofection kit from Lonza following supplier's protocol.

Methods for DNA replication origin firing (6), RNA extraction, generation of cDNA and QPCR have been described earlier (7) and also in the text.

Antibodies and Chemicals: 2A10 antibody was a gift from Arnold Levine. Antibodies against Erk2, chk1, cyclin A, cyclin D2, p73 and p21 were from Santa Cruz Biotechnology, phospho chk1 (p-chk1) from Cell Signaling Technology, and were used following manufacturer's protocol.

IdU was detected by mouse anti-bromodeoxyuridine (Becton Dickinson) primary antibody and Alexafluor 594-conjugated rabbit anti-mouse (Molecular Probes) and Alexafluor 594-conjugated goat anti-rabbit (Molecular Probe) secondary and tertiary antibodies. CldU was detected by rat anti-bromodeoxyuridine (Accurate) primary antibody, and Alexafluor 488-conjugated chicken anti-rat (Molecular Probe) and Alexafluor 488-conjugated goat anti-chicken (Molecular Probes) secondary and tertiary antibodies as described earlier (6).

Ponasterone A (Invitrogen) was used at 1mM concentration for 24 hours as suggested by the supplier. Wortmannin (Upstate Biotechnology) treatments were carried out at a concentration of 50 nM for 4 hours.

Statistical analysis: Distribution of four types of fibers classified as bi-directional origin, origin clusters, elongating forks and terminating forks over different samples was tested for independence with Fisher exact test or by chi-square test in case the computation of Fisher exact test was not feasible due to computer limitations. The null hypothesis of these tests is that the distribution remains independent of the categories, and a significant p value below 0.05 indicates departure from the hypothesis of independence. All statistical analysis was done using the statistical software R v2.13.0.

Supplementary Reference:

1. Mullany, L.K., White, P., Hanse, E.A., Nelsen, C.J., Goggin, M.M., Mullany, J.E., Anttila, C.K., Greenbaum, L.E., Kaestner, K.H. and Albrecht, J.H. (2008) Distinct proliferative and transcriptional effects of the D-type cyclins in vivo. *Cell Cycle*, **7**, 2215-2224.
2. Diao, L. and Chen, Y.G. (2007) PTEN, a general negative regulator of cyclin D expression. *Cell research*, **17**, 291-292.
3. Singh, S., Ramamoorthy, M., Vaughan, C., Yeudall, W.A., Deb, S. and Palit Deb, S. (2013) Human oncoprotein MDM2 activates the Akt signaling pathway through an interaction with the repressor element-1 silencing transcription factor conferring a survival advantage to cancer cells. *Cell Death Differ*, **20**, 558-566.
4. Thelen, M., Wymann, M.P. and Langen, H. (1994) Wortmannin binds specifically to 1-phosphatidylinositol 3-kinase while inhibiting guanine nucleotide-binding protein-coupled receptor signaling in neutrophil leukocytes. *Proceedings of the National Academy of Sciences of the United States of America*, **91**, 4960-4964.
5. Brown, D.R., Deb, S., Munoz, R.M., Subler, M.A. and Deb, S.P. (1993) The tumor suppressor p53 and the oncoprotein simian virus 40 T antigen bind to overlapping domains on the MDM2 protein. *Molecular and cellular biology*, **13**, 6849-6857.
6. Frum, R.A., Deb, S. and Deb, S.P. (2013) Use of the DNA fiber spreading technique to detect the effects of mutant p53 on DNA replication. *Methods Mol Biol*, **962**, 147-155.
7. Frum, R., Ramamoorthy, M., Mohanraj, L., Deb, S. and Deb, S.P. (2009) MDM2 controls the timely expression of cyclin A to regulate the cell cycle. *Mol Cancer Res*, **7**, 1253-1267.
